# Organizational culture and the adoption of anti-smoking initiatives at small to very small workplaces: An organizational level analysis

**DOI:** 10.18332/tpc/100403

**Published:** 2018-12-19

**Authors:** Christine M. Kava, Edith A. Parker, Barbara Baquero, Susan J. Curry, Paul A. Gilbert, Michael Sauder, Daniel K. Sewell

**Affiliations:** 1 Department of Community and Behavioral Health, University of Iowa College of Public Health, Iowa, United States; 2 Department of Health Management and Policy, University of Iowa College of Public Health, Iowa, United States; 3 Department of Sociology, University of Iowa, Iowa, United States; 4 Department of Biostatistics, University of Iowa College of Public Health, Iowa, United States

**Keywords:** smoking, organizational culture, tobacco control, small workplaces

## Abstract

**INTRODUCTION:**

Many workplaces have adopted anti-smoking initiatives to reduce smoking behavior, but small workplaces are less likely to adopt these initiatives. One factor that could influence adoption is organizational culture, defined as the values and assumptions shared by members of an organization. The aim of this study was to examine the types of organizational culture associated with smoking policy strength and adoption of smoking cessation activities at small (20–99 employees) and very small (<20 employees) workplaces. Two study hypotheses were made: An increase in clan culture (characterized by participation in decision-making and human resources development) will be associated with an increase in smoking policy strength (H1) and higher odds of having cessation activities in the workplace (H2).

**METHODS:**

Between June and October 2017, executives and employees coming from small and very small workplaces participated in separate surveys. Executives answered questions about their workplace’s anti-smoking initiatives, while employees completed a 12-item questionnaire about organizational culture. We aggregated employee data to perform linear and logistic regression at the organizational level.

**RESULTS:**

Organizational culture was not significantly associated with smoking policy strength, therefore H1 was not supported. Counter to H2, an increase in clan culture was associated with lower odds of offering smoking cessation activities (OR=0.06; 95% CI: 0.01–0.58).

**CONCLUSIONS:**

We did not find support for the hypothesized relationships. External factors and additional cultural characteristics may explain study findings. Continued research on culture and ways to improve tobacco control within smaller workplaces is needed.

## INTRODUCTION

The current smoking rate among US adults is 16%^1^. In Iowa, this rate is slightly higher at 17%^2^. Smoking is the leading cause of preventable death, responsible for almost half a million deaths each year^3^. To combat the negative consequences associated with smoking, workplaces commonly adopt two types of anti-smoking initiatives: smoke-free policies (rules designed to restrict smoking and protect employees from its harmful effects) and cessation activities (programs designed to reduce smoking). Both have been successful in increasing cessation^4,5^.

Based on previous studies and definitions^6-8^, small workplaces are defined here as employing between 20 to 99 employees, and very small workplaces as employing less than 20 employees. These smaller workplaces are less likely to adopt anti-smoking initiatives, with adoption rates increasing as the size of the workplace increases^7^. To reduce disparities in tobacco control and smoking behavior, it is important to understand factors associated with anti-smoking initiative adoption in smaller workplaces.

One factor to consider is organizational culture, defined as the beliefs and assumptions shared by organizational members^9^. Several frameworks exist to conceptualize culture, including the Competing Values Framework (CVF). The CVF has been extensively used in the field of organizational psychology, it is a theoretically driven framework with strong evidence of instrumental reliability and validity^10,11^, and as a quantitative measure facilitates comparisons of culture across organizations^12^.

The CVF posits that there are two dimensions of organizational effectiveness, represented by four culture types: clan, adhocracy, hierarchy, and market^10^. Clan culture emphasizes participation in decision-making and human resources development. Adhocracy culture emphasizes innovation and entrepreneurship. Hierarchy culture emphasizes structure and standardization. Market culture emphasizes productivity and competition^10,13^. Most organizations have a dominant culture type^10^, however ‘organizations are expected to reflect all four cultures to some degree’, as noted by Helfrich et al.^14^.

Previous studies have found that primary care practices exhibiting a combination of clan and adhocracy cultures were more likely to offer preventative health services, which include services for tobacco^15^. In a similar study examining primary care provider adherence to guidelines for treating tobacco use, clan cultures were more likely to adhere to the 5A guidelines (i.e. ask, advise, assess, assist, arrange) for tobacco treatment compared to adhocracy cultures^16^. Clan culture is also associated with greater workplace practice satisfaction^17^ and well-being of employees^18^.

A majority of studies examining the association between culture and health initiative adoption have been conducted in healthcare settings and focused on service delivery. Given the research described above, it could be argued that workplaces that are higher in clan culture would also be more likely to adopt initiatives for tobacco control. Clan cultures are characterized by a corporate commitment to employees and creation of a ‘humane work environment’^10^. These values may extend to health promotion, with leadership taking steps to adopt initiatives that improve the employees’ well-being and facilitate a healthy working environment.

More broadly, culture plays a prominent role in influencing organizational behavior and decision-making^19^; as described by Tierney^20^, ‘an understanding of culture has become essential for those who seek to understand how to foment change in the organization’. Thus, knowing how culture is related to tobacco control can help guide intervention efforts that enhance cultural characteristics associated with initiative adoption. Additional research is also needed among smaller workplaces, which offer unique challenges and opportunities for health promotion^21^. Previous researchers have called for increased efforts to develop health promotion programs feasible for very small workplaces in particular^6^.

The aim of this study was to examine the types of organizational culture associated with smoking policy strength and adoption of smoking cessation activities at small (20–99 employees) and very small (<20 employees) workplaces, in Iowa. We hypothesized the following relationships:

H1: An increase in clan culture will be associated with an increase in smoking policy strength.

H2: An increase in clan culture will be associated with higher odds of having cessation activities in the workplace.

## METHODS

### Design and recruitment

This study used a cross-sectional survey design. Executives coming from small and very small workplaces in Iowa completed a self-administered online survey about the smoking policies and cessation activities at their workplace. Employees at these same workplaces were then asked to complete a self-administered online survey about their organization’s culture. We identified executives through ReferenceUSA, a business database^22^. To qualify for inclusion, a business had to be listed as being small or very small, verified as active (open), and having an executive e-mail address on file. Only one executive per business was contacted. In the event that multiple executives were listed under a business in the database, we contacted the most recent (current) executive.

Based on these criteria, approximately 40000 small and very small businesses were eligible to participate in the study. We initially sampled 2000 businesses from the ReferenceUSA database using quasi-random sampling techniques (e.g. one of every 20th business selected). Based on low response rates, we sampled additional businesses from the database to ensure that our goal of recruiting at least 60 workplaces with executive and employee data was met (we determined this sample size from a power analysis). In total, we sampled approximately 10500 businesses. We stratified businesses by small and very small workplace classification. After removing duplicate records and workplaces previously contacted for a related study (Kava et al.^23^), 9470 businesses remained and were subsequently contacted.

We conducted recruitment between June and October 2017. We first sent executives an e-mail about the study, which contained a link to the executive online survey. We contacted non-respondents via e-mail and telephone, up to two times, to again request participation. Executives who completed the survey were sent an e-mail containing a link to the employee survey and a request to distribute this e-mail to their employees. Both groups were eligible to win one of three $75 gift cards in separate drawings for their participation. We provided all participants with an information sheet containing elements of consent, which included a description of the study’s purpose and a statement notifying individuals that participation is voluntary. The University of Iowa’s Institutional Review Board approved all study procedures.

### Measures

The dependent variables for this study were *smoking policy strength* and *smoking cessation activities offered.* We obtained information on these variables from executives. The independent variable for this study was *organizational culture*, specifically clan culture. Since culture focuses on shared perceptions we asked employees to describe their culture, afterward aggregating responses to obtain organizational level measures for each culture type. These measures are described in further detail below.

#### Dependent variables

We calculated a smoking policy strength score (range: 0–9) for each workplace by summing up executive responses to questions on the following: outdoor smoking restrictions (1 item), indoor smoking restrictions (1 item), and additional policies related to tobacco (5 items). The item on outdoor smoking restrictions asked executives to indicate whether employees could smoke outside the building and included three response options ranging from ‘Yes, anywhere outside on worksite grounds’ (=0) to ‘No, nowhere on worksite grounds’ (=2). The item on indoor smoking restrictions asked executives to indicate whether employees could smoke inside their workplace building and included three response options ranging from ‘Yes, anywhere inside’ (=0) to ‘No, nowhere inside’ (=2). The 2008 Iowa Smokefree Air Act (ISAA) bans smoking in most enclosed areas within places of employment, but there are noted exceptions to this rule (e.g. smoking is allowed on the gaming floor of casinos)^24^. Thus, we included the item on indoor smoking to ensure that we captured information on all possible workplace smoking policies. Both items were taken from a smoking policy survey developed by the University of Wisconsin’s Comprehensive Cancer Center^25^. We included the five items on additional policies in a checklist and asked executives to indicate whether the policy was present (=1) at their workplace. The items listed went beyond state law requirements, and were included to better assess the effects of culture on policy adoption. An example item is: ‘Employees are not allowed to smoke while working, regardless of where they are located (e.g. in the office, working off-site)’.

To calculate smoking cessation activities, executives indicated from a list of items the smoking cessation activities offered by their workplace. An example item is: ‘Smoking cessation classes or clinics’. We developed these based on qualitative research findings from a related study^23^ and on previous measures^26–28^. We coded workplaces as either having (=1) or not having (=0) cessation activities.

#### Independent variable

To assess *organizational culture*, we asked employees to complete a 12-item questionnaire developed by Yeung et al.^29^, which is based on a validated measure of culture using the CVF^11^. The measure contains three items corresponding to each of the four culture types, and asks employees to indicate the extent to which each item describes their workplace. Response options range from low (=1) to high (=5). An example of one item corresponding to clan culture: ‘This organization is a very personal place. It is like an extended family. People seem to share a lot of themselves’. To create a score for each culture type, corresponding items were averaged together. In the current study, Cronbach’s alpha coefficient ranged from 0.73 (hierarchy culture) to 0.79 (clan culture).

#### Covariates

We adjusted for several workplace characteristics theorized or shown to be associated with tobacco control in previous studies^7,30,31^. Covariate information was primarily obtained from executives, who reported the percentage of their employees that were employed full-time (0–25%; 26–50%; 51–75%; 76–100%), employees’ primary work location (in the building; out in the field), and whether their workplace offered health insurance (yes, no, not sure). We adapted these items from previous studies related to health promotion and smoking cessation^27,30,32^. Executives also reported their workplace industry based on National Institute for Occupational Safety and Health^33^ industry categories.

We used information provided by ReferenceUSA to create two additional variables: workplace size (small; very small) and county urban-rural designation (noncore, micropolitan, small metro, medium metro). County designation categories came from the 2013 National Center for Health Statistics Urban-Rural Classification Scheme for Counties^34^. We collapsed and recoded variables with smaller cell sizes (≤20) based on the distribution of responses: workplace industry (service; non-service), health insurance offered (yes, no/not sure), and percentage of full-time employees (0–75%, >75%).

### Data analysis

We conducted data analysis in Stata 15.0^35^ and R Studio 3.3.2^36^. We first ran descriptive statistics followed by bivariate and multivariable analyses. We used linear regression to test H1 and logistic regression to test H2. Given the study’s broader aim to assess the relationship between organizational culture and anti-smoking initiative adoption, we included all four culture types in the multivariable regression models. To adjust for potential confounders, we also included the covariates described above. The health insurance variable predicted perfectly cessation activities offered (i.e. all workplaces offering health insurance offered cessation activities), so this covariate was excluded from the analysis.

We aggregated employee data by averaging responses to obtain organizational level measures of culture for each workplace. To justify this aggregation, Rho-within-group coefficients for multi-item scales, ρ_wg(j)_, were calculated for each workplace on the four culture types to assess the extent to which employees at the same workplace agreed on culture^37^. Mean ρ_wg(j)_ coefficient values were as follows: 0.76 for clan, 0.73 for adhocracy, 0.73 for hierarchy, and 0.78 for market. These values indicate an adequate level of agreement based on previous recommendations^38^.

## RESULTS

### Participants

In all, 264 executives, each representing one workplace, and 405 employees participated in this study. Not all workplaces had employee data available. Further, a small number of employees (n=9) entered the wrong workplace ID on their survey, which was necessary to link employee responses to those of their executive. In total, executive and employee data were linked at 71 workplaces. Three workplaces employed more than 99 employees, and in two additional cases the executive had recently retired. After excluding executive and employee data from these workplaces, the final sample sizes for this study were 259 executives, 280 employees, and 68 workplaces with both executive and employee data available. The mean number of employees participating at the 68 workplaces was four (range: 1–22).

### Main findings

Table 1 provides information on anti-smoking initiatives in the workplace, based on executive reports (N=259). Almost all executives reported that their workplaces prohibited indoor smoking (97%, n=252). Restrictions on outdoor smoking were less common, with about a third of workplaces (31%, n=79) allowing smoking anywhere outside and 31% (n=81) allowing outdoor smoking in designated areas or times. Approximately 80% of workplaces had additional smoking-related policies. The average smoking policy strength score was 4.84 (SD=1.92) out of nine. Among workplaces offering cessation activities, the most common activity offered was referral for smoking cessation assistance (12%, n=31).

**Table 1:** Descriptive statistics: Anti-smoking initiatives (N=259)^a^

*Variable*	*Mean*	*SD*
**Smoking policy strength score^b^**	4.84	1.92
	***%***	***n***
**Indoor smoking**		
Yes, anywhere inside	0.77	2
Yes, but only in designated areas or times	1.93	5
No, nowhere inside	97.30	252
**Outdoor smoking**		
Yes, anywhere outside	30.62	79
Yes, but only in designated areas or times	31.40	81
No, nowhere outside	37.98	98
**Other smoking policies**		
Employees not allowed to smoke while working, regardless of location	46.67	119
E-cigarette use is restricted	40.00	102
Smokeless tobacco use is restricted	38.04	97
Workplace has written smoking policy	46.27	118
Other policies	9.80	25
None of the above	19.22	49
**One or more activities offered**	23.14	59
**Cessation activity types**		
Referrals for smoking cessation assistance	12.16	31
Incentives for non-smoking or cessation attempts	5.88	15
Self-help materials	8.24	21
Smoking cessation classes or clinics	5.10	13
Lectures or workshops	2.35	6
Other activities	5.88	15
None of the above	76.86	196

a Descriptive statistics calculated using executive data.

b Range: 0–9.

Table 2 describes the organizational characteristics of the sample, with employees at 68 workplaces providing data on culture. Mean culture scores ranged from 3.12 (SD=0.69) for adhocracy culture to 3.81 (SD=0.68) for clan culture. In bivariate analysis (data not shown in table), correlations among the four culture types ranged from 0.136 to 0.645. Culture was not significantly associated with smoking policy strength. Compared to small workplaces, very small workplaces had a lower mean smoking policy strength score (p=0.012). The mean score for clan culture was lower among workplaces offering cessation activities (p=0.000).

**Table 2:** Descriptive statistics: Other organizational characteristics (N=259)^a^

*Variable*	*Mean*	*SD^b^*
**Culture**		
Clan	3.81	0.68
Adhocracy	3.12	0.69
Hierarchical	3.37	0.62
Market	3.72	0.51
	***%***	***n***
**Workplace industry**		
Service	53.91	138
Non-service	46.09	118
**Workplace size**		
Very small (<20 employees)	57.92	150
Small (20–99 employees)	42.08	109
**Employee work location**		
In the building	81.71	210
In the field	18.29	47
**Health insurance offered**		
Yes	69.26	178
No/not sure	30.74	79
**Insurance coverage for cessation medication^c^**		
Yes	27.98	47
No	17.86	30
Not sure	54.17	91
**Per cent of full-time employees**		
0–75%	42.97	110
>75%	57.03	146
**County area designation**		
Non-core	31.35	79
Micropolitan	13.10	33
Small metro	21.83	55
Medium metro	33.73	85

a Culture calculated only among workplaces with employee data available (n=68).

b SD: standard deviation.

c Asked only among employers whose workplace offered health insurance (n=178).

Table 3 shows the results for the multivariable linear regression predicting smoking policy strength. No significant relationship existed between clan culture and smoking policy strength (p=0.806), therefore H1 was not supported. No other culture types were significantly associated with smoking policy strength. After adjusting for workplace characteristics, differences in smoking policy strength by workplace size were no longer significant. A *post hoc* analysis excluding the additional policies in the calculation of the smoking policy strength variable revealed the same findings (data not shown in table). Table 4 shows the findings from the logistic regression predicting smoking cessation activities. As clan culture increased, the odds of having smoking cessation activities decreased (OR=0.06; 95% CI: 0.01–0.58; p=0.015). These findings do not support H2, which theorized that an increase in clan culture would be associated with *higher* odds of offering cessation activities. No other culture types were associated with the likelihood of offering cessation activities.

**Table 3:** Linear regression on smoking policy strength (n=63)

*Variable*	*β*	*SE^a^*	*95% CI^b^*	*p*
**Culture**				
Clan	-0.13	0.53	(-1.21, 0.94)	0.806
Adhocracy	-0.42	0.55	(-1.53, 0.69)	0.448
Hierarchical	0.37	0.48	(-0.60, 1.34)	0.444
Market	-0.20	0.64	(-1.49, 1.10)	0.761
**Workplace industry** (ref: Non-service)				
Service	-0.37	0.58	(-1.54, 0.80)	0.530
**Workplace size** (ref: Very small)				
Small	0.84	0.57	(-0.31, 1.99)	0.148
**Work location** (ref: In the field)				
In the building	0.85	0.70	(-2.25, 0.56)	0.232
**Health insurance offered** (ref: Yes)				
No/not sure	0.97	0.69	(-0.42, 2.37)	0.168
**Per cent full-time employees** (ref: 0–75%)				
>75%	0.75	0.62	(-0.48, 1.99)	0.228
**County designation** (ref: Non-core)				
Micropolitan	0.00	0.74	(-1.49, 1.48)	0.997
Small metro	-0.67	0.80	(-2.27, 0.94)	0.408
Medium metro	0.02	0.67	(-1.32, 1.37)	0.972
**Constant**	6.29	2.35	(1.57, 11.01)	0.010*
**R^2^**	0.18			

a SE: standard error.

b CI: confidence interval.

* p<0.05, **p<0.01, ***p<0.001.

**Table 4:** Logistic regression on smoking cessation activities (n=63)

*Variable*	*OR^a^*	*95% CI^b^*	*p*
**Culture**			
Clan	0.06	(0.01, 0.58)	0.015*
Adhocracy	1.09	(0.13, 9.39)	0.937
Hierarchical	2.71	(0.47, 15.56)	0.263
Market	1.72	(0.22, 13.24)	0.600
**Workplace industry** (ref: Non-service)			
Service	0.48	(0.09, 2.64)	0.398
**Workplace size** (ref: Very small)			
Small	0.68	(0.12, 3.83)	0.659
**Work location** (ref: In the field)			
In the building	0.09	(0.01, 0.79)	0.030*
**Per cent full-time employees** (ref: 0–75%)			
>75%	0.61	(0.10, 3.87)	0.598
**County designation** (ref: Non-core)			
Micropolitan	3.85	(0.36, 40.76)	0.263
Small metro	0.62	(0.04, 9.60)	0.731
Medium metro	2.72	(0.31, 24.08)	0.369

a OR: odds ratio.

b CI: confidence interval.

* p<0.05, **p<0.01, ***p<0.001.

## DISCUSSION

While previous studies have examined organizational culture’s relationship to healthcare outcomes, this study contributes new knowledge on the association between culture and health promotion initiatives related to tobacco. Applying a framework commonly used in organizational psychology (CVF) to gain a greater understanding of tobacco control at smaller workplaces, this study also addressed these relationships within the context of a statewide smoking ban. This study found no significant relationship between clan culture and smoking policy strength. Contrary to expectations, workplaces stronger in clan culture were less likely to offer cessation activities. This is inconsistent with previous studies that have found positive relationships between clan culture and health practices^15,16^.

The lack of support for our study’s hypotheses may be due to several reasons. This study attempted to assess policy strength within the context of the ISAA, a law prohibiting smoking at most workplaces in Iowa^24^. By including questions on initiatives not covered by this Act and creating a total score based on all policy questions, we attempted to gain a better understanding of how culture may influence policy adoption independently. However, given the ISAA’s longstanding presence (10 years) and declining acceptability of smoking in public places, it could be that culture has less of an influence on a workplace’s decision to adopt anti-smoking initiatives. In a related qualitative study assessing the barriers and facilitators to anti-smoking initiative adoption among smaller workplaces^23^, several participants described the ISAA as a reason for policy adoption. Some participants also described wanting to create a healthy work environment, which may indicate that there are additional aspects of organizational culture (e.g. health culture) more important to policy adoption than culture more broadly.

Regarding the significant and negative association between clan culture and cessation activities offered, managers may not perceive a lack of need for activities, particularly if smoking employee rates are low. This idea is supported by qualitative data from a related study, where several participants described this lack of need^23^. At the same time, managers may be willing to offer activities if a perceived need from employees arose. This may be especially true for workplaces strong in clan culture, characterized by employee participation in decision-making processes. Similarly, the supportive and interpersonal nature of clan culture organizations could make managers less willing to provide advice to employees about their health. This unwillingness may stem from manager fears of being viewed as paternalistic, a notion that contrasts with the characteristics of clan culture and a cited barrier to health promotion in previous studies^39^.

Bivariate analysis revealed a lower smoking policy strength score among very small workplaces compared to small workplaces. This relationship became non-significant after accounting for other organizational characteristics, suggesting that differences in these characteristics are important to policy adoption and warrant further attention. Differences by workplace size in smoking cessation activity adoption did not exist, although overall the number of workplaces offering these activities was low (23%, n=59). Given this information, continued efforts to develop tobacco control programs tailored to the environment of smaller workplaces are needed.

Several potential limitations warrant consideration. A lack of generalizability and power to detect small effect sizes may be present, as the response rate and sample sizes for this study were low. While previous studies have used ReferenceUSA and similar databases for recruitment^40-42^, others have called into question the validity of these databases^43^. In the current study, approximately 20% of the recruitment e-mails that were sent to executives came back as undeliverable, suggesting a lack of accuracy that could help to explain the low response rates we observed. Since all workplaces included in this study came from Iowa, they may not be representative of all workplaces in the US. In states with less comprehensive legislation or different social norms related to smoking, these relationships may differ. Nevertheless, our findings are likely to be relevant to other workplaces in similar settings and in states with comparable legislation on tobacco control, and contribute to our limited understanding of these relationships within smaller workplaces.

Executives and employees who did not perceive smoking as an issue at their workplace may have been less likely to participate in the study, leading to non-response bias. After receiving an invitation to participate in the study, some executives indicated that they did not feel their workplace qualified to participate, either because their workplace was smoke-free or because they did not currently employ any smokers. To reduce the potential for bias, these individuals were informed by the first author (CMK) that we were seeking to recruit a diverse range of workplaces. We also adapted our recruitment materials to reflect this point. Lastly, while organizational culture focuses on *shared* assumptions^44^, some of the workplaces included in this study had only one employee participant. Given our focus on smaller workplaces this was not unexpected, but makes it harder to understand culture from a shared viewpoint.

Previous studies have described the challenges associated with measuring culture, a complex construct with multiple dimensions. As described by Scott et al.^45^, ‘A problem with trying to assess highly complex phenomena like culture is that experts rarely agree on which are the essential dimensions to measure’. They go on to suggest assessments of culture via qualitative or mixed methods approaches, which may be better at capturing culture’s complexity. For example, it was speculated earlier that the supportive and interpersonal nature of organizations strong in clan culture may make these workplaces less likely to offer activities for cessation. Taking a qualitative research approach would allow a more detailed exploration of the nuances of clan culture that influence decision-making, leading to a better understanding of how to increase tobacco control within smaller workplaces to improve employee health.

## CONCLUSIONS

The current study found no association between organizational culture and smoking policy strength. In a finding counter to H2, workplaces stronger in clan culture were less likely to offer activities to help their employees quit smoking. State-wide smoking bans and additional cultural characteristics may help to explain organizational decision-making related to tobacco control. After accounting for several organizational features, no differences in anti-smoking initiative adoption by workplace size existed. Adoption of cessation activities was low overall, suggesting a need for increased tobacco control in smaller workplaces.

## CONFLICTS OF INTEREST

The authors declare that they have no competing interests, financial or otherwise, related to the current work. D. K. Sewell reports grants from US Department of Health & Human Services, Centers for Disease Control & Prevention, during the conduct of the study. The rest of the authors also have completed and submitted an ICMJE form for disclosure of potential conflicts of interest.
